# Contribution of Individual and Environmental Factors to Physical Activity Level among Spanish Adults

**DOI:** 10.1371/journal.pone.0038693

**Published:** 2012-06-07

**Authors:** José Antonio Serrano-Sanchez, Angela Lera-Navarro, Cecilia Dorado-García, Juan José González-Henriquez, Joaquin Sanchis-Moysi

**Affiliations:** 1 Departament of Physical Education, Campus Universitario de Tafira s/n, University of Las Palmas de Gran Canaria, Las Palmas de Gran Canaria, Spain; 2 Department of Physical Education, University of A Coruña, A Coruña, Spain; 3 Department of Mathematics, Campus Universitario de Tafira s/n, University of Las Palmas de Gran Canaria, Las Palmas de Gran Canaria, Spain; National Institute of Agronomic Research, France

## Abstract

**Background:**

Lack of physical activity (PA) is a major risk for chronic disease and obesity. The main aims of the present study were to identify individual and environmental factors independently associated with PA and examine the relative contribution of these factors to PA level in Spanish adults.

**Methodology/Principal Findings:**

A population-based cross-sectional sample of 3,000 adults (18–75 years old) from Gran Canaria (Spain) was selected using a multistage stratified random sampling method. The participants were interviewed at home using a validated questionnaire to assess PA as well as individual and environmental factors. The data were analyzed using bivariate and multivariate logistic regression. One demographic variable (education), two cognitive (self-efficacy and perceived barriers), and one social environmental (organized format) were independently associated with PA in both genders. Odds ratios ranged between 1.76–2.07 in men and 1.35–2.50 in women (both *p*<0.05). Individual and environmental factors explained about one-third of the variance in PA level.

**Conclusions/Significance:**

Self-efficacy and perceived barriers were the most significant factors to meet an adequate level of PA. The risk of insufficient PA was twofold greater in men with primary or lesser studies and who are employed. In women, living in rural environments increased the risk of insufficient PA. The promotion of organized PA may be an efficient way to increase the level of PA in the general population. Improvement in the access to sport facilities and places for PA is a prerequisite that may be insufficient and should be combined with strategies to improve self-efficacy and overcome perceived barriers in adulthood.

## Introduction

Physical inactivity is a major risk for mortality and chronic disease in developed societies [1]–[2]. The promotion of physical activity (PA) has been assumed as a worldwide strategy to prevent chronic diseases [3]. Current recommendations for adults include at least 5 days per week of PA of moderate intensity for at least 30 minutes accumulated along the day, in one or several bouts of at least 10 minutes each [4]. However, the PA of a considerable number of adults in western societies is below this minimal threshold for health benefits. In Europe, about two-thirds of adults do not meet the recommendations of PA for health [5], and in Spain, the level of insufficient PA is between 62% and 74% [5]–[6]. The identification of factors influencing PA behaviors can help develop more effective policies and interventions.

Socio-cognitive models derived from the social cognitive theory (SCT) [7] have been a common approach to the study of PA behavior. Various socio-cognitive factors, such as perceived barriers, self-efficacy, social support, and outcome expectations, have been associated with PA [8]–[10]. SCT emphasizes the importance of social influences and cognitions for being physically active. From the nineties, the ecologic perspective has consolidated and contributed to extend the focus beyond psychological factors to integrate influences from the environment and emphasize physical and social influences [11]–[14]. The ecologic model assumes that PA behavior is influenced by an interaction of different levels of individual, social, and environmental factors [11], [15]. Physical and social environments play a central role in PA behavior, together with cognitive and biological factors [11]. Hence, the built environment and places for PA could be independently associated with PA and may play a role as important as self-efficacy or other socio-cognitive factors to explain the variance in PA. Studies examining the joint influence of cognitive and physical environment factors on walking showed that the contribution of a supportive physical environment was similar to the contribution of positive cognitions, after controlling for social and demographics factors [16].

The ecological perspective has been proposed to assess the correlates of PA [17]–[18]; however, most of the studies were carried out in North America and Australia. There are no studies on Spanish adults that utilize a multilevel perspective to assess the correlates of PA participation. Using a multilevel model with Spanish university students, Molina-Garcia et al. tested the influence of psychological and environmental factors concerning active commuting to university [19]. The results showed that access to private motorized transport (negatively), psychosocial barriers (negatively) and walking facilities (positively) were associated with active transport more strongly than self-efficacy, whereas socio-economic status, access to public transport and distance to university was not associated with active transport [19]. Cultural and environmental background (i.e., neighborhood characteristics, availability of facilities) could be different in Spain from those in Australia or North America, and the relation of individual and environmental factors with PA could be potentially partially different [20]–[21].

The simultaneous evaluation of behavioral determinants from multiple levels has been proposed to provide insight into the relative importance of personal, social and physical environmental influences on PA behavior [22]. The present study includes well known individual, social and environmental correlates of PA to assess their relative contribution to recommended PA level. Otherwise, multilevel statistical techniques also has been proposed to examine the relationship between environment and PA behavior because they enable the analysis of not only the relative contribution of explanatory variables, but also the “cross-level” interactions [23]–[24]. Individual behaviors could be affected by geographical influences [25], town-size [26] and other population-level influences [20]. To isolate this potential bias, we stratified the sample by geographical zone and town-size to guarantee the presence and heterogeneity of all strata, using the town as first sampling unit. We then used a two-level multivariate analysis with towns as second level to test the random effects on individual level of PA. This approach has the advantage in that it allows examination of the degree of variation present between and within sampling cluster [27], helping to detect potential neighborhood self-selection bias [28].

Thus, the aims of the present study were to determine the associations between the recommended level of PA in Spanish adults and two sets of factors: (1) individual (demographic, personal, and cognitive) and (2) environmental (social and physical).

## Methods

### Sample and data collection

A cross-sectional sample of the adult population (18–75 years old) residing in Gran Canaria (Canary Islands, Spain) participated in this study. The sample size (*n* = 3000) was stratified according to geographical location (five strata) and town size (six strata). In each stratum, data from population census were classified according to age and gender. Eighty-two towns were randomly selected with representations of all strata. In each town, the number of houses proportional to the number of interviews was randomly selected. In each home, one adult with independent life was interviewed between May and June 2004 by a professional interviewer who received 20 hours of training. When the person with the selected profile was not at home, a second visit was programmed (the reply ratio was 73.2%, *n* = 2.196). If the interviews could not be carried out, the contiguous household was included in the study. A total of 150 participants were interviewed 2–4 weeks later to evaluate reliability. The study was performed in accordance with the Helsinki Declaration of 1975, last modified in 2000. The bioethics committee of the University of Las Palmas de Gran Canaria approved the study. The participants were informed of the objectives, requested for their written consent to participate anonymously, and interviewed in their homes.

### Measurements

#### Physical activity

PA was assessed using the Minnesota Leisure Time Physical Activity Questionnaire (MLTPAQ). MLTPAQ was selected because of its concurrent validity in Spanish adults (*r* = 0.39–0.57 against cardiorespiratory fitness) [29]–[30]. The interviewees were asked about the number of days and duration of their participation in 71 recreational and occupational PA in the last week. The participants were classified into two groups according to public health recommendations [4], [31]. The *sufficiently active* group met one of the three following conditions: (1) 3 days/week (d/w) and 20 minutes/day (min/d) of vigorous PA (>6 MET), (2) 5 d/w and 30 min/d of moderate PA (3.5–6 MET), or (3) 5 d/w and 30 min/d of either combination of moderate and vigorous PA with an energy expenditure of at least 600 MET-minutes/week [32]; otherwise, the participants were classified as *insufficiently active*.

#### Individual variables

Individual variables comprised two sets: (1) demographic and personal, and (2) cognitive. *Demographic and personal* variables included eight characteristics previously identified [33] as PA correlates: sex (male and female), age (continuous, 18–75 years old), education (primary or lesser, secondary, and university), employment status (employed and unemployed), household income (five groups with intervals of 6000 €/year), perceived health (5-point scale from “poor” to “excellent”), smoking (coded into 10 cigarettes per day), and body mass index (BMI) obtained by asking the height and weight of the participants. The BMI computed through self-reported measures underestimated the objective BMI by 0.56 kg/m^2^ (95% CI, −0.71 to −0.41); nevertheless, both measurements demonstrated high correlation (*r* = 0.95), which validated their epidemiological use [34].

*Cognitive variables* included three correlates derived from SCT applied to PA behavior [35]: self-efficacy, perceived barriers, and outcome expectations. The internal consistency (Cronbach's α) and reliability (test-retest correlation coefficient) were tested. Self-efficacy items were selected from previously published research [36]–[37]. Participants were asked for the degree of confidence to perform moderate PA 5 days/week and 30 minutes/day with four perceived barriers (lack of time, tiredness, mood disturbance, and boredom), using a five-point scale from 1 (not at all confident) to 5 (very confident). Items were averaged to obtain an index for self-efficacy (α = 0.83, *r* = 0.79). The outcome expectation items were based on previous studies analyzing the beliefs of outcomes as consequence of performing regular physical activity [38]. We reduced the items to three empirical dimensions that have been identified in factorial analyses: physical health, mental health and social-recreational [39]–[40]. Participants were asked by the degree of agreement on the improvement of physical health, state of mind, and sociability if they performed regular PA using a four-point scale (1, strongly disagree; 2, disagree; 3, agree; and 4, strongly agree). The index of outcome expectations was calculated using the average of the items (α = 0.82, *r* = 0.87). Thirteen perceived barriers of PA (i.e., lack of time, fear of injury, getting tired) were identified in the literature [41]–[42] and included in the present study. Perceived barriers were assessed by asking the participants about the frequency of each barrier using a five-point scale (from 1 [never] to 5 [very often]). As the internal consistency was low (α = 0.43), the perceived barriers were analyzed separately. Five perceived barriers were not associated with PA (bad weather, unsafe, not being in good health, discouraged by others, and taking care of people) and were excluded. To assess the effect of accumulation of perceived barriers, the average from the other eight perceived barriers was calculated to obtain an index of perceived barriers (*r* = 0.77).

#### Environment variables

Physical environment variables comprise availability of facilities, perceived access to facilities and eight perceived physical characteristics of the neighborhood. The questions used were similar to those reported in previous studies [43]–[44]. The participants were asked for the presence of sport facilities, parks, and walking trails in their neighborhood (yes or no) (*r* = 0.92). Perceived access to spaces for PA was evaluated by asking the participants if they had access to places indoor, outdoor, or both in their neighborhood for PA purposes (*r* = 0.94). Eight physical and social environmental characteristics of the neighborhood, included in the tables, were evaluated by asking the participants about their presence (yes or no) (*r* = 0.89).

Social environment was assessed through social support, modeling and format of participation in PA. Social support questions were previously tested [45]. Selected items used in previous research [45]–[46] were included in the present study. Social support was assessed using four questions related to having received motivational reinforcement and social accompaniment for PA (from friends and family) with a four-point scale (1, strongly disagree; 2, disagree; 3, agree; and 4, strongly agree). An index of social support was generated calculating the average of the items (α = 0.74, *r* = 0.82). Social modeling was evaluated with regard to family, friends, and neighbors [47] with three questions: “A lot of your [family/friends/neighbors] are physically active. Would you say that this statement is 1) not at all true, 2) somewhat true, 3) true, or 4) very true” (α = 0.66). The question about neighbors was excluded to generate an average index of modeling (α = 0.75, *r* = 0.89). Format of PA was assessed using two questions: the first was to inform about the presence of a monitor throughout the PA sessions (yes or no), and the second was to inform about the enrollment of the participant as a member or user of a club or gym where PA took place (yes or no). The variable was coded into two categories: 1, not organized (neither of the two conditions), and 2, organized (one of the two conditions) (*r* = 0.93). Information about the population size of the town was obtained from census and coded as <10,000 and ≥10,000 inhabitants.

### Data analyses

Differences in the PA level between sampling clusters were tested with a multilevel analysis, introducing the towns into a random effect model in two-level (individual and town). The variance partition coefficient (VPC) was not significant in women (VPC = 0.5%, p>0.05), whereas in men, it was significant (p<0.05) but with low relevance (VPC = 3.2%). We tested the design effect, showing values of 1.55 and 1.08 for men and women respectively. The examination of multilevel assumptions suggested that a single-level analysis would be sufficient. Simple and multiple logistic regressions with recommended level of PA as binary dependent variable were performed. Correlation matrix did not show values higher than 0.30 between independent variables, except for the index of social support and the index of outcome expectations (*r* = 0.34, *p*<0.001). Both variables were found to be associated with PA in bivariable analyses and were retained in the multivariable model. Two models of logistic regressions, namely, bivariable and multivariable, both segmented by gender, were essayed. The multivariable model included the two sets of variables indicated earlier (individual and environmental), as follow: age, BMI, perceived health, indexes of self-efficacy, perceived barriers, outcome expectations, modeling and social support were introduced as continuous variables, and the remaining individual (education, employment status and smoking) and environmental variables (availability of facilities, access to facilities, eight neighborhood characteristics, format of physical activity and town size) were introduced as categorical variables. Household income was excluded from the final model because of lack of association with PA and moderate association with education. Multivariable analysis accurately classified 74% of the male participants (75% sensitivity, 72% specificity) and 71% of the female participants (72% sensitivity, 70% specificity). Hosmer and Lemeshow test (H–L test) was used to assess the goodness of fit, which showed values of 0.41 in men and 0.38 in women, allowing rejection of the hypothesis stating the inadequate fit of the model. Odds ratios and their 95% CIs were used as association measures between level of PA and individual variables. Significant differences were assumed when *p*<0.05. A step-by-step logistic regression was performed to assess the relative contribution of the examined variables on the PA level. The percentage of change in the coefficient of determination, pseudo-R^2^ Nagelkerke (R^2^_N_), was observed to quantify the relative contribution of the covariates on the variance of PA. The R^2^_N_ satisfied the six criteria used to the measures based on R^2^ to inform about the explained variance when the same sample and predictors were used [48]. The data were analyzed using SPSS (v. 18.0, IBM).

## Results

### Basic characteristics of the participants

Primary or lesser education was the most common response (>50%) (Table 1), and 61.5% of men and 37.3% of women were employees. Household incomes were similarly distributed across categories, with a slight higher frequency of participants situated in lower levels of income (53.9% of men and 59.1% of women below 18,000 €). One-third of both the genders resided in towns with less than 10,000 inhabitants. More than 50% of male and female participants were overweight (BMI≥25 kg/m^2^). The perceived health was reported as poor or fair by 22.9% of men and 29.9% of women. Smoking showed a prevalence of 40.7% in men and 25.9% in women, most in the higher category (≥20 cigarettes). The prevalence of sufficient PA was 54.8% and 50.1% for male and female participants, respectively.

**Table 1:** Basic characteristics of adults participants in Gran Canaria Physical Activity Study.

	Male			Female		
	n	%	% Active	n	%	% Active
**Total**	1505	100	54.8	1495	100	50.1
**Age** a						
18–29 years	487	32.4	65.1	474	31.7	55.7
30–44 years	486	32.3	49.6	484	32.4	50.8
45–59 years	307	20.4	45.9	292	19.5	50.3
60–75 years	225	15.0	55.6	245	16.4	37.6
**Education**						
Primary or lesser	856	56.9	49.4	874	58.5	45.3
Secondary	478	31.8	60.3	442	29.6	56.6
University	169	11.2	65.9	176	11.8	57.4
**Employment status**						
Employed	925	61.5	51.0	558	37.3	47.7
Unemployed	577	38.3	61.3	934	62.5	51.6
**Household Income**						
<12,000 €	333	22.1	50.8	375	25.1	47.3
12,000–17,999 €	478	31.8	51.6	509	34.0	48.1
18,000–23,999 €	466	31.0	52.3	413	27.6	49.1
≥24,000 €	203	13.5	51.5	182	12.2	51.3
**Size of town**						
<10,000 inhabitants	511	34.0	53.0	496	33.2	47.5
≥10,000 inhabitants	994	66.0	57.4	999	66.8	53.7
**Perceived health**						
Poor	57	3.8	47.4	84	5.6	33.3
Fair	287	19.1	49.1	364	24.3	48.9
Good	770	51.2	55.1	771	51.6	51.6
Very good	228	15.1	59.1	169	11.3	52.1
Excellent	160	10.6	59.9	106	7.1	53.8
**Smoking** b						
None	897	59.6	59.3	1108	74.1	50.1
<10 cigarettes	110	7.3	66.4	79	5.3	53.2
10–19 cigarettes	138	9.2	47.8	116	7.8	53.4
≥20 cigarettes	353	23.5	42.8	190	12.7	46.8
**BMI** c						
18.5 to 24.99 kg/m^2^	657	43.7	58.9	746	49.9	54.0
25–26.99 kg/m^2^	324	21.5	55.3	232	15.5	54.5
27–29.99 kg/m^2^	284	18.9	55.0	238	15.9	47.0
≥30 kg/m^2^	212	14.1	43.4	247	16.5	42.1

a Age, Mean ± SD = 40.1±15.8 years-old;

b Smoking, 18±11.3 cigarrettes;

c BMI, 25.6±4.5 kg/m^2^; *% Active*, percentage of participants meeting public health recommendations of PA.

### Associations between individual variables and physical activity

Table 2 presents the relation between individual variables and the level of PA. After adjustment, university education was the only individual-demographic variable associated with PA level in both genders (*p*<0.05), when compared with the unadjusted model. Employed men were negatively associated with PA level (OR = 0.48; 95% CI, 0.37–0.69). Male smokers also showed about 14% higher risk than nonsmokers of failure to reach the recommended levels of PA for each increase of 10 cigarettes per day (OR = 0.88; 95% CI, 0.80–0.98).

**Table 2:** Associations of individual factors with the recommended level of physical activity.

	Unadjusted				Adjusted^a^			
	Male		Female		Male		Female	
	OR	(95% CI)	OR	(95% CI)	OR	(95% CI)	OR	(95% CI)
**Demographic and personal**								
Age^b^	0.99	(0.98–0.99) *	0.98	(0.97–0.99)	0.99	(0.98–1.01)	0.99	(0.98–1.00)
Education								
Primary or lesser	1.00	(ref)	1.00	(ref)	1.00	(ref)	1.00	(ref)
Secondary	1.55	(1.24–1.95) *	1.57	(1.25–1.98) *	1.27	(0.94–1.71)	1.19	(0.84–1.56)
University	1.97	(1.39–2.80) *	1.62	(1.17–2.25) *	2.07	(1.30–3.30) *	1.35	(1.03–1.65) *
Employment status								
Unemployed	1.00	(ref)	1.00	(ref)	1.00	(ref)	1.00	(ref)
Employed	0.60	(0.48–0.79) *	0.85	(0.67–1.07)	0.48	(0.37–0.69) *	0.81	(0.60–1.09)
Smoking^c^	0.80	(0.74–0.87) *	0.98	(0.89–1.08)	0.88	(0.80–0.98) *	0.99	(0.87–1.13)
BMI^d^	0.95	(0.92–0.97) *	0.97	(0.95–0.99) *	0.98	(0.95–1.01)	0.99	(0.96–1.03)
Perceived health	1.16	(1.04–1.29) *	1.15	(1.03–1.28) *	1.00	(0.87–1.16)	0.95	(0.76–1.11)
**Cognitive** e								
Self-efficacy	1.92	(1.77–2.08) *	1.86	(1.72–2.01) *	1.89	(1.67–2.11) *	1.73	(1.57–1.90) *
Outcome Expectations	1.50	(1.23–1.84) *	1.42	(1.16–1.73) *	0.93	(0.74–1.17)	1.09	(0.80–1.47)
Barriers	0.32	(0.24–0.42) *	0.36	(0.28–0.47) *	0.48	(0.35–0.65) *	0.40	(0.32–0.53) *
Lack of time	0.91	(0.86–0.97) *	0.84	(0.79–0.89) *	0.87	(0.81–0.94) *	0.87	(0.81–0.94) *
No motivation	0.73	(0.65–0.80) *	0.68	(0.62–0.75) *	0.71	(0.61–0.83) *	0.72	(0.64–0.82) *
Do not like exercise	0.67	(0.58–0.77) *	0.71	(0.63–0.80) *	0.75	(0.61–0.92) *	0.86	(0.74–0.99) *
Be tired	0.80	(0.74–0.86) *	0.76	(0.70–0.82) *	0.89	(0.79–0.99) *	0.94	(0.84–1.05)
No energy	0.77	(0.68–0.89) *	0.72	(0.65–0.79) *	1.03	(0.85–1.25)	0.98	(0.85–1.13)
Get exercise at job	0.77	(0.70–0.84) *	0.83	(0.75–0.93) *	0.91	(0.81–1.03)	0.93	(0.81–1.06)
Afraid of injury	1.05	(0.94–1.18)	0.87	(0.77–0.97) *	1.18	(0.99–1.40)	1.03	(0.85–1.18)
Self-conscious	0.85	(0.66–1.09)	0.81	(0.68–0.96) *	1.00	(0.72–1.42)	0.92	(0.74–1.14)

OR = Odds ratio to be sufficiently active; 95% CI = 95% confidence interval; * p<0.05;

a Logistic Regression adjusted by *age; education; employment status; smoking; BMI; perceived health; indexes of self-efficacy, outcome expectations, barriers, social support and modelling; format of PA; availability of sport facilities, walking trails and parks; perceived access to facilities for PA; 8 characteristics of neighborhood; and town size*.

b for each increase of 1 year;

c for each increase of 10 cigarretes;

d for each increase of 1 kg/m^2^;

e all cognitive variables as ordinal scales.

All cognitive variables were associated with PA in unadjusted analyses, but after adjusting, self-efficacy and perceived barriers were found to be significantly associated with PA in both genders (*p*<0.05) (Table 2). For each increase of 1 point in self-efficacy's scale, the likelihood to meet the recommended level of PA increased by 89% and 73% in men and women, respectively (*p*<0.05). The index of perceived barriers and some individual perceived barriers were negatively associated with PA. For each increase of 1 point in the index of perceived barriers, the risk of failure in meeting appropriate PA level increased by 108% in men (OR = 0.48; 95% CI, 0.35–0.46) and 117% in women (OR = 0.46; 95% CI, 0.35–0.61).

### Associations between environmental variables and physical activity

Before adjustment, access to facilities, organized format, and some neighborhood characteristics were associated with PA level in men and women (all *p*<0.05) (Table 3). Availability of facilities was not associated with PA. After adjustment, only the organized format was associated with PA level in both genders, with 76% and 111% increase in the odds to meet the recommended level of PA in men and women, respectively (*p*<0.05). In women, perceived access to a combination of indoor and outdoor spaces (OR = 1.47; 95% CI, 1.36–2.28) and modeling (OR = 1.30; 95% CI, 1.05–1.60) remained significant after adjustment. The town size also showed significant differences of PA among women. Those living in towns <10,000 inhabitants showed a lower participation in PA as recommended (OR = 0.62, *p*<0.05). None of the environmental attributes of neighborhood remained significantly associated after adjustment.

**Table 3:** Associations of environmental factors with the recommended level of physical activity.

	Unadjusted				Adjusted^a^			
	Male		Female		Male		Female	
	OR	(95% CI)	OR	(95% CI)	OR	(95% CI)	OR	(95% CI)
**Social environment**								
Social Support	1.56	(1.35–1.80) *	1.28	(1.11–1.49) *	1.19	(0.95–1.49)	0.97	(0.85–1.23)
Modeling	1.38	(1.22–1.59) *	1.34	(1.17–1.53) *	1.05	(0.86–1.26)	1.30	(1.05–1.60) *
Format of physical activity								
No organized	1.00	(ref)	1.00	(ref)	1.00	(ref)	1.00	(ref)
Organized	1.99	(1.42–2.79) *	2.55	(1.67–3.90) *	1.76	(1.17–2.66) *	2.11	(1.28–3.50) *
**Physical environment**								
**Availability of facilities**								
None	1.00	(ref)	1.00	(ref)	1.00	(ref)	1.00	(ref)
At least one	1.36	(0.98–1.90)	1.05	(0.75–1.46)	1.30	(0.87–1.95)	0.80	(0.53–1.19)
Complementary^b^	1.00	(ref)	1.00	(ref)	1.00	(ref)	1.00	(ref)
Sport facilities	1.14	(0.92–1.41)	1.12	(0.91–1.38)	0.89	(0.66–1.21)	0.94	(0.70–1.25)
Walking trails	1.04	(0.84–1.27)	0.96	(0.79–1.18)	0.90	(0.68–1.19)	0.94	(0.72–1.23)
Parks	1.13	(0.90–1.41)	1.08	(0.86–1.35)	1.20	(0.89–1.61)	0.92	(0.69–1.22)
**Access to facilities**								
None	1.00	(ref)	1.00	(ref)	1.00	(ref)	1.00	(ref)
At least one	1.70	(1.37–2.11) *	1.97	(1.58–2.45) *	1.34	(0.98–1.87)	1.47	(1.36–2.28) *
Indoor and outdor	1.69	(1.33–2.14) *	1.98	(1.55–2.52) *	1.28	(0.89–1.83)	1.52	(1.05–2.20) *
Indoor only	1.90	(1.31–2.77) *	1.96	(1.44–2.66) *	1.41	(0.85–2.31)	1.32	(0.92–2.03)
Outdoor only	1.61	(1.18–2.20) *	1.94	(1.37–2.76) *	1.48	(0.96–2.30)	1.40	(0.87–2.30)
**Neighborhood characteristics**								
Complementary^c^	1.00	(ref)	1.00	(ref)	1.00	(ref)	1.00	(ref)
Noysi streets	1.34	(1.08–1.66) *	1.39	(1.13–1.72) *	1.23	(0.91–1.73)	1.07	(0.78–1.48)
Pollution	1.31	(1.05–1.65) *	1.44	(1.16–1.80) *	1.29	(0.91–1.85)	1.34	(0.95–1.89)
Enjojable scenery	1.26	(1.01–1.58) *	1.08	(0.87–1.35)	1.06	(0.77–1.47)	1.01	(0.73–1.39)
Unattended dogs	1.16	(0.94–1.45)	1.10	(0.89–1.38)	0.90	(0.68–1.19)	1.14	(0.87–1.50)
Hills	0.83	(0.73–1.02)	0.85	(0.69–1.05)	0.91	(0.67–1.24)	0.97	(0.72–1.31)
Heavy traffic	1.23	(0.99–1.50)	1.12	(0.91–1.37)	1.08	(0.82–1.44)	1.07	(0.81–1.43)
Streetlights	0.91	(0.60, 1.40)	0.80	(0.53–1.18)	1.04	(0.60–1.80)	0.73	(0.44–1.23)
Sidewalks	1.01	(0.77–1.32)	0.90	(0.68–1.19)	0.89	(0.62–1.28)	0.67	(0.45–1.08)
**Size of town**								
<10 000 inhabitants	1.00	(ref)	1.00	(ref)	1.00	(ref)	1.00	(ref)
≥10 000 inhabitants	1.20	(0.97–1.47)	1.28	(1.04–1.57) *	1.05	(0.74–1.48)	1.62	(1.14–2.28) *

OR = Odds ratio to be sufficiently active; 95% CI = 95% confidence interval;

* p<0.05;

a Logistic Regression adjusted by *age; education; employment status; smoking; BMI; perceived health; indexes of self-efficacy, outcome expectations, barriers, social support and modelling; format of PA; availability of sport facilities, walking trails and parks; perceived access to facilities for PA; 8 characteristics of neighborhood; and town size*.

b no availability of the specific type of facility;

c Ausence of the specific attribute.

### Relative contribution of individual, and environmental variables to physical activity

Table 4 shows the explained variance (pseudo-R^2^_N_) and the percentage of contribution to pseudo-R^2^_N_ for each variable and set of variables. Cognitive factors contributed to coefficient of determination by 66.1% (R^2^_N_ = 0.25) and 73.2% (R^2^_N_ = 0.23) in men and women, respectively. Demographic factors contributed 22.4% in men (R^2^_N_ = 0.086) and 10.9% in women (R^2^_N_ = 0.035). The contribution of environmental factors were 11.5% (R^2^_N_ = 0.044) and 15.9% (R^2^_N_ = 0.051) in men and women, respectively. Individual factors with the highest contribution to explained variance were self-efficacy (60.4% in men and 63.6% in women), perceived barriers (5.5% in men and 9.7% in women), employment status (7.8% in men and 3.4% in women), and format of PA (4.9% in men and 7.5% in women).

**Table 4:** Percentage of the determination's coefficient explained by individual and environmental variables.

	Male		Female	
	pseudo-R^2^	%	pseudo-R^2^	%
**Individual**	0.340	88.5	0.270	84.1
**Demographic and personal**	0.086	22.4	0.035	10.9
*Age*	*0.015*	*3.9*	*0.018*	*5.6*
*Education*	*0.017*	*4.4*	*0.005*	*1.6*
*Employment status*	*0.030*	*7.8*	*0.011*	*3.4*
*Smoking*	*0.017*	*4.4*	*0.000*	*0.0*
*BMI*	*0.005*	*1.3*	*0.001*	*0.3*
*Perceived health*	*0.002*	*0.5*	*0.000*	*0.0*
**Cognitive**	0.254	66.1	0.235	73.2
*Self-efficacy*	*0.232*	*60.4*	*0.204*	*63.6*
*Expectations*	*0.001*	*0.3*	*0.000*	*0.0*
*Barriers*	*0.021*	*5.5*	*0.031*	*9.7*
**Environment**	0.044	11.5	0.051	15.9
**Social environment**	0.022	5.7	0.028	8.7
*Social support*	*0.003*	*0.8*	*0.001*	*0.3*
*Modeling*	*0.000*	*0.0*	*0.003*	*0.9*
*Format of physical activity*	*0.019*	*4.9*	*0.024*	*7.5*
**Physical environment**	0.022	5.7	0.023	7.2
*Availability of facilities*	*0.003*	*0.8*	*0.001*	*0.3*
*Perceived access to facilities*	*0.003*	*0.8*	*0.004*	*1.2*
*Neighborhood characteristics*	*0.015*	*3.9*	*0.013*	*4.0*
*Size of town*	*0.001*	*0.3*	*0.005*	*1.6*
**Total**	0.384	100	0.321	100

## Discussion

### Individual factors

We found that almost half of the men and half of the women did not meet the recommended level of PA for health, accounting for recreational and occupational PA of ≥3.5 MET (equivalent to walk for pleasure) [49]. Our values are lower than those reported from Madrid (Spain), showing that 65% of men and 79% of women were below the recommended level of PA [50]. This discrepancy may be because the Madrid's study only accounted for recreational PA [50]. Men and women with primary or less education, paid workers and male smokers, and rural women were the groups with major risk of insufficient PA. Work commitments have been shown as the most important perceived barrier for PA in Spanish adults (37%) in a previous European research [51]. Similarly, we observed a lower level of PA among employed participants, independent of other individual and environmental conditions. Among Japanese adults, employed men also showed a lower level of PA, when compared with the unemployed ones [52]. On the other hand, the present findings support that lower PA levels are widespread in women living in rural environments [53]–[55] and that education level is consistently associated with PA [33]. However, other studies showed that adults with university education were not associated with the recommended level of PA [5], [56], even negatively associated with meeting a high level of PA [56]. In these studies, PA at work was included to estimate the prevalence of sufficient PA [5], [56], which may explain the differences compared with studies analyzing only recreational PA [50], [57]. It has been reported that recreational PA tended to be lower in those who were more physically active at work [58], and higher education has been associated with more time sitting [5]. Age and BMI were not independently associated with PA level. It seems that age tends to lose association with PA in the presence of other demographic and socio-cognitive factors [18], [53]. Indeed, it has been reported that individuals above retirement age (65–69 years old) are physically more active than those in the preceding age group (60–64 years old) [59]. With respect to BMI, the lack of independent association with PA observed in the present study could be attributed to cognitive factors. Blanchard et al. [12] showed that obese adults tend to have a lower self-efficacy than overweight and normal adults. Furthermore, self-efficacy was associated with PA in the three weight groups, but the association was lower in obese than in the other two groups [12]. As BMI moderated the associations of PA with self-efficacy, the authors recommended including BMI as a covariate when examining socio-cognitive and PA relations [12].

Among the individual factors analyzed, self-efficacy and perceived barriers were the most important. The risk of not achieving the recommended level of PA increased twofold for each increase of 1 point in the scale of index of perceived barriers. Other studies including individual, social and environmental factors have reported perceived barriers as a strong independent correlate of recommended PA [43], [60], being higher in women [53], [61]. Some perceived barriers (lack of time, I do not like exercise and no motivation) had similar importance for PA level than those showed in other studies with population from the United States [43] and Japan [53].

A similar but positive association was observed for self-efficacy, with slightly lower size effects. These results are congruent with those of previous studies, showing self-efficacy as a consistent cognitive predictor of PA [10], [53], [61]–[63]. In the present study, outcome expectations were not independently associated with PA. This lack of associations could be attributed to an insignificant influence of outcome expectations on PA level in adults. One in-depth study that focused on social-cognitive determinants of PA in adults also documented no associations between outcome expectations and PA level [8]. In adults 60 years old and older, expectations regarding health benefits was associated with PA in a bivariate analysis but not in multivariate analysis with other psychosocial and perceived environment variables [59]. The strong associations of self-efficacy and perceived barriers on PA suggest that interventions designed to increase PA should be addressed to increase self-efficacy and overcome perceived barriers. Self-efficacy is sensible to social influences, and likely, interventions should focus on creating supportive environments and observational learning in the home and neighborhood settings [8], [60]–[61].

### Environmental factors

The most important social factor was the organized format, which was independently associated with PA level in both genders. It seems that the social context of PA, such as collective and affiliated, has a positive impact on the level of PA. A possible explanation could be that adults assisted through organized PA sessions receive more social influences than adults involved in nonorganized PA [64]. Organized participation could gather several sources of social influences, which, when combined, may increase positive cognitions to perform regular PA. Previous studies have shown that participants in organized activities were 2.5 times more likely to meet the recommended level of PA than participants who are not organized (OR = 2.45; 95% CI, 1.86–3.22) [15]. Our results also concur also with other multivariate studies reporting similar size effects for membership of sporting and recreational clubs [65].

Some multivariate research found independent associations of social support with attaining the recommended level of PA [15], [18]; however this was not the case for the current study. The lack of independent associations of social support could be because of a lesser importance on PA once the behavior is established [46], [66], as well as a differential association depending on the type of PA performed. Walking was the most prevalent PA among study participants, including occupational walking which may be less sensitive to emotional and social reinforcement compared with other recreational and more intense PAs [18]. Otherwise, associations of social support with overall PA might be indirect. It has been suggested that self-efficacy mediates the associations of other socio-cognitive factors with PA [59]. In partial agreement with our results, McNeill et al. [63] reported an indirect effect of social support on PA, and Anderson et al. [8] found that social support influenced PA indirectly through self-efficacy. Other studies also reported that social support was inconsistently associated with PA [53], [61].

Modeling may have a greater influence in women than in men on the recommended level of PA [67]. In support, in the present study modeling was found to be independently associated with PA in women. This suggests that interventions in women should consider including observational learning and normative changes oriented to remove physical and normative barriers in open public spaces, as components to increase PA for health.

In the reviewed literature, we found a discrepancy with regard to the influence of the availability of sport facilities, walking trails, and parks on the PA level. The present study focused on perceived presence and access and found no associations between these factors and PA level after adjustment. Similarly, other studies have failed to find an association between free access to sport facilities and PA using measures derived from geographical information system (GIS) [68], as well as the availability of gyms and swimming pools with their use [69]. Therefore, availability of sport facilities itself does not appear to be a determinant to increase the level of PA [53], [70]. Nevertheless, the availability of sport facilities appears to be a necessary condition, although not sufficient, because their absence or living farther from facilities and/or difficulty in walking trails could reduce the level of PA [71]–[73]. A study conducted on Spanish adults found that more than 50% of users of sport facilities reside at a distance below 1.3 km [74]. In contrast, Duncan et al. [18] found a positive association between distance to parkland and the level of PA in Australian adults using GIS-derived measures. The authors argued that active adults probably had a greater ability to overcome perceived barriers of distance compared with less active population and that the simple proximity could be less important than skills and abilities to engage in moderate-to-vigorous PA [18]. This explanation is congruent with a study showing that the relation between perceived physical environment and PA is mediated by self-efficacy [75]. Lower self-efficacy also has been linked to a lower perceived access to sport facilities [76]–[77]. In the present study, perceived access to sport facilities was found to be associated with PA level in both genders before adjustment, but this was not the case after adjusting for other demographic, socio-cognitive, and environmental factors. Only female participants with perceived access to a combination of indoor and outdoor sport facilities showed positive associations with PA as recommended.

### Relative importance of individual, and environmental factors in physical activity

Cognitive factors were found to be the major contributor to the explained variance of the recommended level of PA, explaining two-thirds of the determination coefficient in men and three-fourths in women, whereas demographic and environmental factors were observed to account for the rest. Particularly, self-efficacy and perceived barriers (cognitive), together with organized format (social-environment) as well as employment status and education (socio-demographic), accumulated about three-fourths of the determination coefficient in both genders. Nevertheless, the amount of variance explained by the whole model was about one-third. As logistic regression tends to underestimate the variance, when compared with lineal regression [78], it is expected to find differences when comparing studies using the coefficient of determination. Using linear regression, De Bourdeaudhuij et al. [79] showed that in Portuguese adults, socio-cognitive factors explained all the variance (R^2^ = 0.41) in moderate-to-vigorous PA in leisure time. Other studies using structural equations have observed that socio-cognitive variables may explain 46–52% of the variance in PA [8], [80].

In the present study, environmental factors accounted for about 6–9% of the explained variance in the recommended PA level in both genders. Similar percentage of explained variance for environmental factors also has been shown in other studies [79], [81]. However, the relative importance of neighborhood environment may be different for walking activities, when compared with the overall PA [82]. Some environmental factors, such as land-use mix [77], [79], street connectivity [83], and proximal footpaths [18], could be as important as socio-cognitive factors in physical activities such as walking and active transport. With regard to the recommended PA level, the presence of and access to supportive infrastructure and the quality of neighborhood had a low contribution in the present study. Only social environment (organized format) for both genders and rural condition for women had independent associations with the level of PA.

### Limitations

Several limitations should be mentioned. First, cross-sectional analysis does not allow the determination of the direction and causality of associations, which may be achieved with longitudinal or intervention studies. Nevertheless, a review of environmental determinants of PA has shown that longitudinal and cross-sectional studies report similar results [83]. Second, PA was assessed using a questionnaire that has lower precision compared with other methods (i.e., accelerometer). In turn, the questionnaire has the advantage of reaching larger populations and registering specific dominions of PA (i.e., recreational, occupational). Another limitation is the simplicity of socio-cognitive variables that do not account for the overall complexity of social influences. In the present study, limited time to conduct the interviews determined the total number of questions. The study population is only a representative of the Island of Gran Canaria, which is culturally and ethnically comparable with the rest of the Spanish population, with the advantage of favorable and controlled climatic conditions (annual temperatures between 18 and 24°C, 21 rainy days per year, and 65–70% environmental humidity levels). Finally, other perceived environmental attributes of places, such as attractive, convenient, or geographical location, were not observed in the present study, and there is some evidence that quality and attractiveness are important factors in attracting more people toward PA in open public spaces [16], [84].

### Conclusions

Half of the women and almost half of the men did not meet the recommended level of moderate-to-vigorous PA for adults. Self-efficacy and perceived barriers were the most important contributors to meet an adequate level of PA. The risk of insufficient PA was twofold greater in employed men and those with primary or lesser education. In women, the associations of education and work activity with PA level were more attenuated, but rural conditions increased the risk of insufficient PA. The promotion of organized PA may be an efficient way to increase the level of PA in the general population. However, improvement in the access to sport facilities or places for PA is a prerequisite that may be insufficient and should be combined with strategies to improve self-efficacy and overcome perceived barriers in adulthood. Further studies are needed to assess the moderate effect of cognitive factors on relationship between physical and social environment with individual PA behavior using conceptual and methodological multilevel techniques for controlling potential cross-level interactions.
